# Expression of NF-κB p65 phosphorylated at serine-536 in rectal cancer with or without preoperative radiotherapy

**DOI:** 10.2478/v10019-011-0030-7

**Published:** 2011-09-22

**Authors:** Andreas Lewander, Jinfang Gao, Gunnar Adell, Hong Zhang, Xiao-Feng Sun

**Affiliations:** 1 Department of Oncology, Institute of Clinical and Experimental Medicine, University of Linköping, Linköping, Sweden; 2 Department of Oncology, Karolinska University Hospital, Stockholm, Sweden; 3 Department of Biomedicine, University of Skövde, Sweden

**Keywords:** NF-κB, serine-536, radiotherapy, rectal cancer, immunohistochemistry, recurrence, prognosis

## Abstract

**Background:**

In the present study, we investigated NF-κB p65 phosphorylated at Serine-536 (phosphor-Ser536-p65) in rectal cancer and its relationship to preoperative radiotherapy (RT), clinicopathological variables and biological factors.

**Patients and methods:**

Expression of phosphor-Ser536-p65 was examined by using immunohistochemistry in 141 primary rectal cancers, 149 normal mucosa specimens and 48 metastases in the lymph nodes, from rectal cancer patients who participated in a Swedish clinical trial of preoperative RT.

**Results:**

The expression of phosphor-Ser536-p65 in the cytoplasm increased from normal mucosa to primary tumour (p<0.0001, for both the group that did and the group that did not received RT). The expression did not further increase from primary tumour to metastasis in either group (p>0.05). Expression of phosphor-Ser536-p65 was positively related to, or tended to be related to, the expression of tumour endothelium marker 1 (TEM1, p=0.02), FXYD-3 (p=0.001), phosphatase of regenerating liver (PRL, p=0.02), p73 (p=0.048) and meningioma associated protein (MAC30, p=0.05) in the group that received RT but there were no such relationships in the group that did not received RT (p>0.05). The expression of phosphor-Ser536-p65 was not related to clinicopathological factors including survival (p>0.05).

**Conclusions:**

The increased expression of phosphor-Ser536-p65 may be involved in rectal cancer development. After RT, phosphor-Ser536-p65 seems to be positively related to the biological factors, which associated with more malignant features of tumours. However, phosphor-Ser536-p65 was not directly related to the response of RT based on recurrence and survival.

## Introduction

Nuclear factor-kappaB (NF-κB) is responsible for expression by regulating many genes for immune response, cell adhesion, differentiation, proliferation, angiogenesis and apoptosis. The function of NF-κB is inhibited by binding to NF-κB inhibitory proteins, and imbalance of NF-κB and its inhibitors has been associated with development of tumours and other diseases.^1^–^3^ Five members of the NF-κB family have been found in human cells, RelA (p65), p105/p50, p100/p52, RelB and c-Rel. The most common form in human cells is p65/p50 heterodimer. The regulation of the NF-κB protein family is very important. Upon activating signals the inhibitory proteins are degraded and the protein translocates into the nucleus where it exert its effect. The regulation also occurs at the posttranslational level, where protein phosphorylation of the different subunit is one very important mechanism of regulation. Several different phosphorylation sites on the subunits have been discovered. An important site of phosphorylation of p65 subunit is at Serine-536 (phospho-Ser536-p65), and this phosphorylation is involved in regulation of transcriptional activity, nuclear localisation and protein stability.^1^,^2^,^4^,^5^

It has also been shown that in some tumours NF-κB activation can enhance radiosensitivity.^6^,^7^ Preoperative radiotherapy (RT) is today a standard treatment for rectal cancer patients in Sweden and other countries.^8^ It has been shown to increase survival of the patients.^9^,^10^ In the present study, we investigated whether the phospho-Ser536-p65 was related to response of RT in rectal cancer patients who received or did not receive RT, and whether there were any relationships of the phospho-Ser536-p65 with clinicopathological variables and biological factors.

## Patients and methods

### Patients

This study included the patients with rectal adenocarcinoma from the Southeast Swedish Health Care region that participated in a Swedish clinical trial of preoperative RT between 1987 and 1990.^9^ Surgical specimens were obtained by either rectal amputation or anterior resection from 141 patients. The mean age at diagnosis was 66 years (range 36–85). The mean follow-up time was 83 months (range 0–193). Seventy-nine patients had surgery alone. Sixty-two patients were randomised to preoperative radiotherapy, receiving 25 Gy in 5 fractions over a median of 6 days (range 5–12). Surgery was performed after a median of 3 days (range 1–13) after radiotherapy. The characteristics of the patients and tumours are given in Table 1.

The data regarding expression of tumour endothelium marker 1 (TEM1, unpublished data), FXYD-3 (also known as MAT-8), phosphatase of regenerating liver (PRL, also known as PTP4A3, protein-tyrosine phosphatase), p73 and meningioma associated protein (MAC30) on the same material used as in the present study, determined by immunohistochemistry, were taken from previous studies performed at our laboratory.^11^–^14^ The number of the patients listed in Table 2 was less than the number of the patients mentioned in the materials of the present study due to available numbers of the previous cases^11^–^14^, which matched, with the present study. The immunohistochemical staining for those factors was performed on the normal mucosa, primary tumour and metastasis in the lymph nodes from both the non-RT and RT groups.

### Immunohistochemistry

Five-micrometer sections were deparaffinised in xylene and rehydrated in graded ethanol. As the method for antigen retrieval we used was high-pressure cooking in 0.01 M Tris-EDTA buffer (pH 9.0). The sections were heated to 125°C for 30 sec and then cooled to 90°C for 10 sec, the sections were then kept in the buffer till room temperature. The sections were incubated with 3% H_2_O_2_-methanol for 20 min and washed with phosphate-buffered saline (PBS, pH 7.4). After that the sections were incubated with rabbit anti-phospho-Ser536-p65 antibody (phospho S536, ab28856, Abcam, Cambridge, MA) at 20 mg/ml in antibody diluent (Dako, Carpinteria, CA) overnight, followed by rinsing with PBS. The antibody binds specifically to the Ser536-phosphorylated form of p65 and does not cross-react with non-phosphorylated p65 or any other members of the NF-κB family. Subsequently, the sections were incubated with a goat anti-rabbit/ mouse, coupled with peroxidase provided by the Dako ChemMate EnVision Detection Kit (Dako) for 25 min, and washed with PBS. The peroxidase reaction, using 3,3′-diaminobenzidine tetrahydrochloride, was performed (Dako) for 8 min. Sections known to stain positively were included as positive controls. The negative controls used PBS instead of the primary antibody. In all staining procedures, the positive controls showed clear staining, and there was no staining in the negative controls.

The sections were microscopically examined and scored independently by Lewander A and Gao J without any information on the clinicopathological data. The slides were initially classified as weak including negative (<5% of positive cells) and strong staining in the cytoplasm of normal epithelial cells, and tumour cells and metastasis irrespectively of the percentage of positive cells. To avoid artificial effects, cells in areas with necrosis, with poor morphology, or in the margins of sections were excluded from the analysis.

### Statistical analysis

The significance of the difference in phospho-Ser536-p65 expression between normal mucosa, primary tumour and metastasis was tested by Chi-square and McNemar methods. The relationships between phospho-Ser536-p65 expression and clinicopathological/biological variables were examined by Chi-square method, and the relationships to survival were tested by using Cox’s proportional hazard model. Survival curves were calculated by using the Kaplan-Meier method. Two-sided p values of <0.05 were considered statistically significant.

## Results

### Phospho-Ser536-p65 expression in the cytoplasm of normal mucosa, primary tumour and metastasis in the lymph node

**FIGURE 1:**
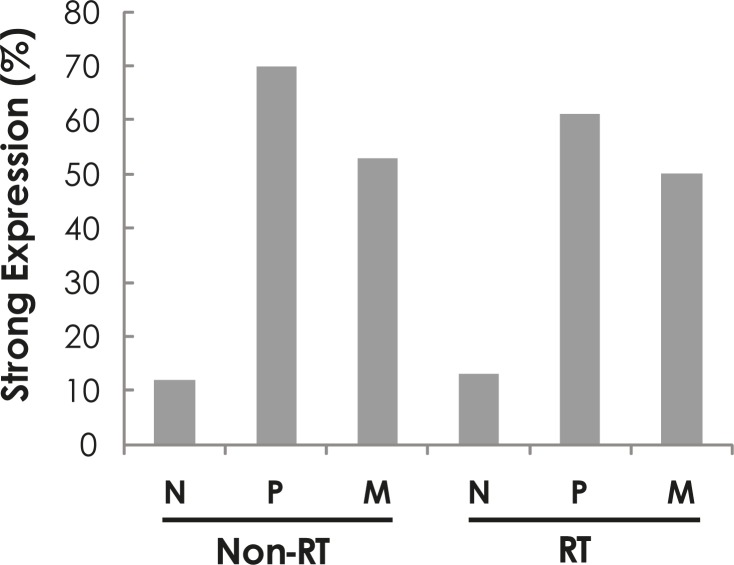
Frequency of strong phospho-Ser536-p65 expression in normal mucosa (N), primary tumour (P) and metastasis in the lymph nodes (M) in non-radiotherapy (non-RT) and radiotherapy (RT).

When we compared staining intensity of phospho-Ser536-p65 expression in the cytoplasm of normal mucosa, primary tumour and metastasis in the lymph node we found significantly more samples with the strong staining of phospho-Ser536-p65 expression in primary tumour than in normal mucosa in the both non-RT and RT groups (p<0.0001 for both Chi-square and McNemar tests for both non-RT and RT groups, Figure 1). There was no significant difference between primary tumour and metastases in either non-RT or RT groups (p>0.05, Figure 1).

We compared phospho-Ser536-p65 expression before and after RT and found there were no differences in normal mucosa (p=0.06), primary tumour (p=0.30) as well as metastases (p=0.81) with chi-square test.

**FIGURE 2:**
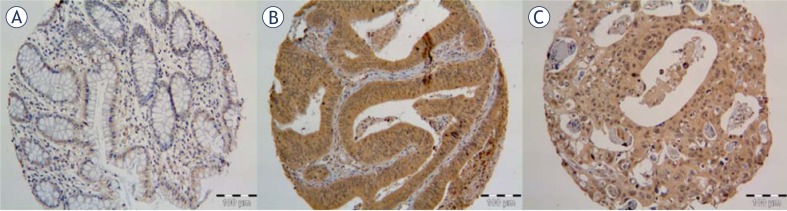
The expression of phospho-Ser536-p65 was weak in normal mucosa (A) and strong expression in primary tumour (B) and metastases in the lymph node (C).

Figure 2 shows phospho-Ser536-p65 expression in normal mucosa, primary tumour and surgical specimens) and metastases in the lymph node. There was weak phospho-Ser536-p65 expression in normal mucosa, while strong expression in the cytoplasm of primary surgical and metastatic tumours.

### Phospho-Ser536-p65expression in the cytoplasm in relation to clinicopathological and biological factors

We compared the expression of phospho-Ser536-p65 expression with the expression of TEM1, FXYD3, PRL, p73 and MAC30 (Table 2). Phospho-Ser536-p65 expression was positively related to or tended to be positively related to TEM1 (p=0.02), FXYD-3 (p=0.001), PRL (p=0.02) and p73 (p=0.048) and MAC30 (p=0.05) in the RT group. However in the non-RT group, there were no such relationships (p>0.05, Table 2).

We analysed the relationship of phospho-Ser536-p65 expression in the cytoplasm of primary tumour with clinicopathological variables and did not find any statistically significant relationship of phospho-Ser536-p65 expression with gender, age, differentiation, stage, local/distant recurrence and survival in the two sub-groups of non-RT and RT, or in the whole group of the patients (p>0.05, data not shown).

## Discussion

In this study we examined materials from rectal cancer patients included in the Swedish rectal cancer trial of preoperative RT^9^, *i.e.*, the patients divided into two groups, one that received and one that did not received preoperative RT.

When we compared staining intensity of phospho-Ser536-p65 expression in the cytoplasm of normal mucosa, primary tumour and metastasis in the lymph node we found significantly more samples with strong staining in primary tumour than in normal mucosa in either the non-RT or RT group. There was no significant difference between primary tumour and metastases in either the non-RT or RT group. Others have found similar results, that NF-κB is upregulated in tumour cells compared with the corresponding normal cells in previous studies. Lind *et al.* used electrophoretic mobility shift assay (EMSA) technique and demonstrated that NF-κB in primary tumour was greatly increased compared with adjacent normal tissue from the same patients.^15^ Yu *et al.* examined the expression of NF-κB p65 by using a monoclonal antibody against NF-κB p65 in normal colorectal mucosa, colorectal adenomas and colorectal adenocarcinomas, and showed that NF-κB p65 expression was significantly increased from normal mucosa to adenoma and to adenocarcinoma, furthermore the expression was increased with the transition from low to moderate and to high dysplasia of adenoma.^16^ Our previous study in colorectal cancer by immunohistochemistry using the same antibody, showed primary tumour had stronger phospho-Ser536-p65 expression than normal mucosa but had no difference between primary tumours and metastases in the lymph node (unpublished data). Taken together, these results indicate that the NF-κB p65 may play a role in earlier development of colorectal cancer.

In the same materials used here we have previously studied expression of TEM1 (unpublished data), FXYD3 (9), PRL (11), p73 (10) and MAC30 (12). We found that phospho-Ser536-p65 expression was positively related to TEM1, FXYD-3, PRL, p73 and MAC30 in tumours that received RT, however there were no such relationships in the non-RT group. TEM1 was expressed on periendothelial mural cells (*i.e.,* pericytes) and activated tumour fibroblasts, probably played a role in the tumour vasculature.^17^–^19^ In our previous study we found TEM1 expression in the stroma increased from normal mucosa to primary tumour both in the non-RT and RT group. In the RT group, TEM1 expression in the stroma significantly increased from Dukes’ A to B-D. FXYD-3 is an 8-kDa trans-membrane protein and acts as a chloride channel or chloride channel regulator.^20^ FXYD-3 is overexpressed in several types of cancers including colorectal cancer.^11^,^20^,^21^ In our previous study, we found that FXYD-3 expression in the primary tumours was, or tended to be increased compared with normal mucosa regardless of RT. Furthermore in the RT group, strong FXYD-3 expression alone or combined with PRL was related to an unfavourable prognosis independent of both, the TNM stage and tumour differentiation, which are important prognostic factors.^22^ In tumours with strong FXYD-3 expression, there were less tumour necrosis and a trend of increased incidence of distant metastasis after RT. None of these effects was seen in the non-RT group.^11^ PRL was identified as an important protein in the metastatic process of colorectal cancer. The PRL family consists of three members, PRL-1, -2, and -3. PRL-3, as a tyrosine phosphatase, may play critical roles in the regulation of cellular growth and cell cycle.^23^,^24^ We earlier found that PRL expression was increased from normal mucosa to primary tumour. In the RT group, strong PRL expression was related to distant recurrence and poor survival, independent of both stage and differentiation, but not in the non-RT group. Overexpression of p73 protein has also been correlated with a poor prognosis in colorectal, hepatocellular and breast cancers.^25^,^26^ In the same material, we earlier found that p73 was overexpressed in rectal cancer compared with normal mucosa. The patients with p73-over-expressing tumours tended to have a higher local recurrence after RT compared to non-RT cases.^12^ MAC30 mRNA is expressed in the foetal liver, but not in the adult liver, suggesting a possible role in growth and differentiation of liver.^27^,^28^ The expression of MAC30 is stronger in breast, stomach and colorectal cancers than the corresponding normal tissues^14^,^29^,^30^, indicating that MAC30 may act as an oncogene in the cancers and might play a role in tumour development and aggressiveness. Why the relationship of phospho-Ser536-p65 expression with TEM1, FXYD-3, PRL, p73 and MAC30 in the RT cases but not in the non-RT cases in this study? One possible speculation is due to the effect of RT, namely, RT resulted in these proteins being more active, temporarily or permanently, and the cells tried to survive. The results may raise a notion that one should consider the targets of RT and the checkpoints controlling the pathways which those factors were involved in. The impact of RT on phospho-Ser536-p65 protein needs to be further investigated in a larger number of patients.

In conclusion, the positive expression of phos-pho-Ser536-p65 may be involved in rectal cancer development. After RT, the expression of phospho-Ser536-p65 was positively related to the biological factors which associated with more malignant features of tumours. However, we did not find that the NF-κB protein was directly related to the response of RT based on local/distant recurrence and survival.

## Figures and Tables

**TABLE 1:** Characteristics of patients and rectal cancers

**Characteristics**	**Non-Radiotherapy**		**Radiotherapy**	

	**No. (%)**		**No. (%)**	
**Gender**				
Male	45	(57)	39	(63)
Female	34	(43)	23	(37)
**Age** (years)				
≤66	29	(37)	23	(37)
>66	50	(63)	39	(63)
**Stage**				
I	22	(28)	16	(25)
IIA	18	(23)	22	(36)
IIIA	9	(11)	0	
IIIB	12	(15)	16	(25)
IIIC	14	(18)	3	(5)
IV	4	(5)	5	(8)
**Differentiation**				
Well	5	(6)	4	(6)
Moderately	56	(71)	40	(65)
Poorly	18	(23)	18	(29)
**Numbers of tumours**				
Single	68	(86)	51	(82)
Multiple*	9	(11)	11	(18)
Unknown	2	(3)	0	
**Surgical type**				
Rectal amputation	42	(53)	22	(35)
Anterior resection	37	(47)	40	(65)
**Resection margin**				
Tumour free	75	(95)	59	(95)
Tumour involved margin	4	(5)	3	(5)
**Distance to anal verge** (cm)				
Mean	7.3		8.8	

* Other colorectal cancer or other type of tumour besides the present rectal cancer.

**TABLE 2:** Expression of NF-κB phosphorylated at Serine-536 in relation to biological factors expressed in rectal cancer

	**Non-radiotherapy**				**p-value**	**Radiotherapy**				**p-value**
	**Weak (%)**		**Strong (%)**			**Weak (%)**		**Strong (%)**		
**TEM1**										
Weak	9	(39)	14	(61)	0.43	10	(59)	7	(41)	0.02
Strong	13	(30)	31	(70)		8	(25)	24	(75)	
**FXYD3**										
Weak	13	(45)	16	(55)	0.08	13	(68)	6	(32)	0.001
Strong	10	(25)	30	(75)		7	(21)	27	(79)	
**PRL**										
Weak	12	(34)	23	(66)	1.00	11	(50)	11	(50)	0.024
Strong	9	(33)	18	(67)		5	(19)	21	(81)	
**p73**										
Weak	7	(29)	17	(71)	0.55	9	(56)	7	(44)	0.048
Strong	16	(36)	28	(64)		8	(27)	22	(73)	
**MAC30**										
Weak	9	(31)	20	(69)	0.73	9	(53)	8	(47)	0.05
Strong	13	(35)	24	(65)		8	(25)	24	(75)	
